# A new species of the genus *Seticornuta* Morley (Hymenoptera, Ichneumonidae, Metopiinae) from South Korea

**DOI:** 10.3897/zookeys.478.9048

**Published:** 2015-01-28

**Authors:** Jin-Kyung Choi, Janko Kolarov, Jong-Wook Lee

**Affiliations:** 1 Department of Life Sciences, Yeungnam University, Gyeongsan, 712-749, Korea; 2 Faculty of Pedagogy, University of Plovdiv, 24 Tsar Assen Str., 4000 Plovdiv, Bulgaria

**Keywords:** Eastern Palaearctic, Oriental, *Seticornuta koreana*, South Korea, taxonomy, key

## Abstract

Old World species of the genus *Seticornuta* Morley are reviewed. Seven species of this genus were recorded worldwide, but only one species, *Seticornuta albopilosa* (Cameron), was known from the Old World. Here, we report one new species, *Seticornuta koreana* **sp. n.**, from South Korea, and redescribe the other known Old World species, *Seticornuta albopilosa*, with photographs.

## Introduction

*Seticornuta* Morley is a rarely collected genus belonging to the subfamily Metopiinae. It is a small group, consisting of seven known extant species worldwide. Until now only one species, *Seticornuta albopilosa* (Cameron, 1907), has been recorded from the Oriental and Eastern Palaearctic regions. *Seticornuta apicalis* (Cresson, 1864) and *Seticornuta terminalis* (Ashmead, 1896) are only known to occur in North America, *Seticornuta altamirae* Gauld & Sithole, 2002 and *Seticornuta cryptica* Gauld & Sithole, 2002 in Costa Rica, *Seticornuta cortesi* Porter, 1998 in Chile, and *Seticornuta jacutinga* Araujo & Penteado-Dias, 2012 from Brazil. In this study, we describe a new species, *Seticornuta koreana* Lee & Choi, sp. n., from South Korea. We also provide a redescription and photos of *Seticornuta albopilosa*, and a key to the Old World *Seticornuta* species.

## Materials and methods

Materials used in this study were collected by sweeping and Malaise trapping, after which they were deposited in the animal systematic laboratory of Yeungnam University, Gyeongsan, South Korea (YNU). Specimens were examined using an AxioCam MRc5 camera attached to a stereo microscope (Zeiss SteREO Discovery. V20; Carl Zeiss, Göttingen, Germany), processed using AxioVision SE64 software (Carl Zeiss), and optimized with a Delta imaging system (i-solution, IMT i-Solution Inc. Vancouver, Canada) Measurements are reported for the holotype followed by variation in other specimens in brackets.

Abbreviations are as follows: NHM, The Natural History Museum, London, United Kingdom; ZSI, Zoological Survey of India, Calcutta, India; GG, Gyeonggi-do; CN, Chungcheongnam-do; GB, Gyeongsangbuk-do; GN, Gyeongsangnam-do.

## Results

### Genus Seticornuta

Taxon classificationAnimaliaHymenopteraIchneumonidae

Morley, 1913

Megatrema Cameron 1907: 468. Type species: *Megatrema albopilosa* Cameron, by monotypy. Junior homonym of *Megatrema* Leach
Seticornuta Morley 1913: 310. Type species: *Seticornuta albicalcar* Morley (= *albopilosa* Cameron), by original designation.

#### Diagnosis.

*Seticornuta* species are moderate sized about 5–12 mm and generally blackish or black and yellow. Mandibles not twisted; labrum exposed when mandibles closed (Fig. 3A). Lower face moderately convex; upper face produced upwards into a small tooth between bases of antennae, but projection does not reach frons. Posterior part of head moderately to steeply declivous behind posterior ocelli (Fig. 3C, F). Propodeum moderately short, rather flat and more abruptly declivous posteriorly, with very strong median longitudinal carinae. Tergite I short with strong lateral and median longitudinal carinae. The New World genus *Leurus* is similar but the mandibles of *Seticornuta* species are slenderer and flanged (Gauld et al. 2002). New World species differ from Old World in their smaller size, lower number of antennal flagellomeres and the weakly concave apical margin of the clypeus.

#### Key to Old World species of the genus

***Seticornuta***

**Table:** 

1	Antennal scape and basal flagellomeres reddish brown (Figs 2, 3D–F). Line of combined face and mandible almost square (Fig. 3D). Mesoscutum rounded in lateral view (Fig. 3F). Areola and basal area not separated by carina (Fig. 3J). Spiracles of propodeum linear. Median longitudinal carinae convergent apically (Fig. 3J). Areolet of fore wing with short stalk (stalk shorter than vein 2rs-m; Fig. 3H)	***Seticornuta albopilosa* (Cameron, 1907)**
–	Antennal scape and flagellomeres black (Figs 1, 3A–C). Line of combined face and mandible rounded (Fig. 3A). Mesoscutum steeply sloping (Fig. 3C). Areola and basal area separated by carina (Fig. 3I). Spiracles of propodeum oval. Median longitudinal carinae parallel (Fig. 3I). Fore wing with long stalked areolet (length of stalk as long as vein 2rs-m; Fig. 3G)	***Seticornuta koreana* Lee & Choi, sp. n.**

**Figure 1.**
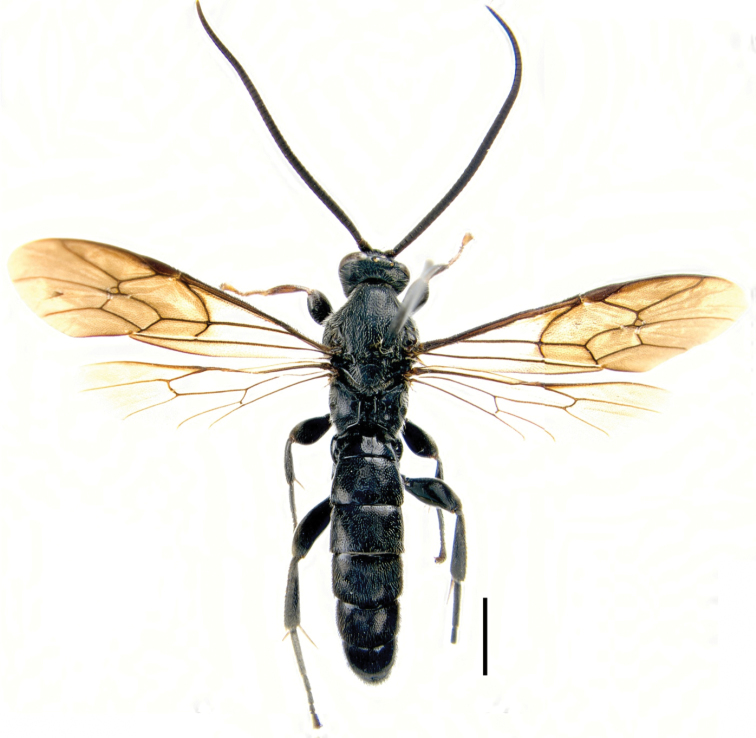
(Holotype) Habitus of *Seticornuta koreana* Lee & Choi, sp. n. Scale bar = 2.0 mm.

**Figure 2.**
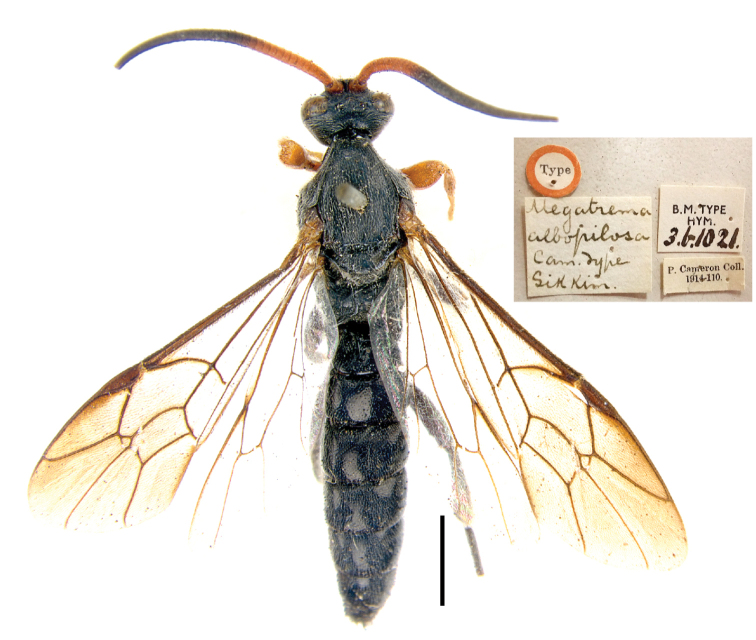
(Holotype) Habitus of *Seticornuta albopilosa*. Scale bar = 2.0 mm.

**Figure 3.**
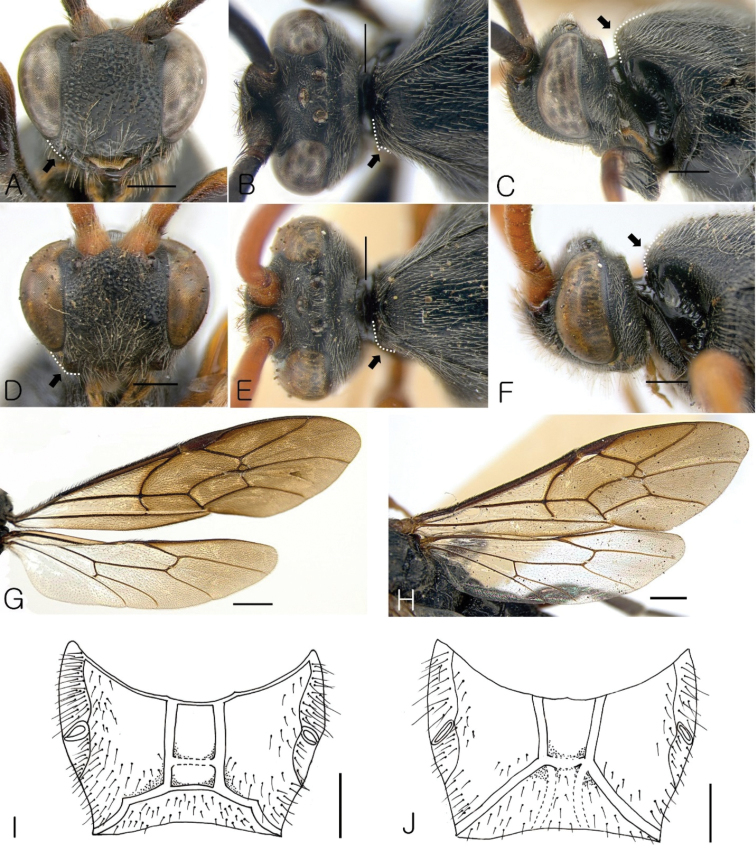
(**A–C, G, I**) *Seticornuta koreana* Lee & Choi, sp. n.; **A** Head in frontal view **B** Head in dorsal view **C** Head in lateral view **G** Wings **I** Propodeum (**D–F, H, J**) *Seticornuta albopilosa* **D** Head in frontal view **E** Head in dorsal view **F** Head in lateral view **H** Wings **J** Propodeum. Scale bars: **G, H** = 1.0 mm; **A–F, I, J** = 0.5 mm.

### Seticornuta koreana

Taxon classificationAnimaliaHymenopteraIchneumonidae

Lee & Choi sp. n.

http://zoobank.org/86CB4DCD-65BE-4E28-98F1-137AF4848425

#### Holotype.

**Female.** Fore wing 9.1 mm (8.3–9.5 mm), body 11.3 mm (10.5–12.0 mm), ovipositor sheath 1.0 mm (1.0–1.1 mm) long.

#### Color.

Black. Wings dark brown; front surfaces of fore tibia and tarsus as well as partial apical lower part of fore femur reddish brown.

#### Morphology.

***Head*.** Face swollen, 1.3 times as long as wide in front view; head strongly narrowed behind eye in dorsal view. Occipital carina strong dorsally and laterally, obsolescent ventrally. Frons smooth with moderately coarse and dense punctures. Inner margin of eye indented a little above antennal socket. Diameter of lateral ocellus equal to shortest distance between ocellus and eye. Flagellum thickened in basal half, tapered to apex, with 47–50 flagellomeres. First flagellomere1.4 times as long as wide, next flagellomere transverse, and last 6–7 flagellomeres square. Clypeus not separated from face. Combined face and clypeus almost square and lower part of gena below the eye rounded in frontal view (Fig. 3A, dotted line and arrow). Face very coarsely and densely punctate, with distance between punctures 0.3 times as long as their diameter, clypeus with more sparse punctures. Upper half of face strongly protruding in profile (Fig. 3C). Mandible except teeth with moderately dense and coarse punctures. Lower tooth of mandible shorter than upper tooth. Malar space 0.33 times as long as basal width of mandible.

***Mesosoma*.** Flattened, 1.7 times as long as high in lateral view. Pronotum smooth, impunctate, protruding into an acute tooth laterally, in dorsal view (Fig. 3B, dotted line and arrow). Epomia weak. Mesoscutum elongate, with sparse punctures, anteriorly narrowly rounded in lateral view (Fig. 3C, dotted line and arrow), notaulus weak. Scutellum flat, without lateral carinae. Epicnemial carina strong, reaching subtegular ridge. Mesopleuron strongly swollen, with sparse punctures in front half. Submetapleural carina lobed. Metapleuron glabrous, impunctate, its lower front ridge strongly projecting as a tooth above mid coxa. Fore wing with petiolate areolet, length of stalk as long as 2rs-m. Hind outer angle of second discoidal cell sharp. Fore wing vein cu-a curved (Fig. 3G). Hind wing with 11 distal hamuli. Nervellus inclivous, intercepted distinctly below middle. Legs very stout. Hind femur coarsely punctate, 2.5 times as long as wide. Ratio between lengths of hind tarsomeres 63:21:18:11:20. Spurs of mid tibia of equal length. Tarsomeres 2–4 of fore leg shorter than wide. Longer spur of hind tibia 0.5 times as long as basitarsus. Propodeum with very strong median longitudinal and apical transverse carinae (Fig. 3I). Combined basal area and area superomedia with parallel sides. Basal area separated from area superomedia by weak carina in some specimens (Fig. 3I). Costula absent. Lateral area punctate except in anterior inner part. Propodeal spiracle 3.0 times as long as wide, joining pleural carina.

***Metasoma*.** Strongly punctate on second to fourth tergites, more weakly on successive following tergites. Median dorsal carinae of first tergite very strong, extending to 2/3 its length. Second tergite 0.7 times as long as wide. Epipleurum of second tergite 1.5 times as long as wide. Ovipositor sheath with long hairs. Metasoma covered with rather long hairs.

#### Male.

Flagellum with 45 flagellomeres. Other characters as in female.

#### Material examined.

**Holotype:** female, South Korea CN, Daejeon-si, Dong-gu, Daejeon University, 16 May–5 June 2006, J.W. Lee (YNU).

**Paratypes.** 1 male (YNU), South Korea, Seoul, Achasan, 24 August 1980, K.S. Jang; 1 female (YNU), GG, Yongmunsa, 1 September 1980, K.S. Jang; 1 male (YNU), GG, Sudong, Chukryeongsan, 28 September 1980, J.I. Kim; 1 female (YNU), South Korea CN, Buyeo-gun, Gyuam-myeon, Sumok-ri, 1-15 June 2005, J.W. Lee; 1 female (YNU), CN, Daejeon-si, Dong-gu, Daejeon University, 16 May-5 June 2006, J.W. Lee; 1 male (YNU), GB, Cheongdo-gun Unmun-myeon, Sinwon-ri, Unmunsan, Unmunsa, 17 July 1989, J.W. Lee; 1 male (YNU), GN, Jinju-si, Gajoa-dong, 19-23 June 1989, J.G. Kim

#### Distribution.

South Korea.

#### Host.

Unknown.

#### Etymology.

The specific name is derived from South Korea, the country of the type specimens.

#### Remarks.

The new species is distinguished from *Seticornuta albopilosa* by the following characters: antenna entirely black (reddish brown in basal half in *Seticornuta albopilosa*) and propodeum with areola separated from area basalis (areola merged with area basalis in *Seticornuta albopilosa*).

### Seticornuta albopilosa

Taxon classificationAnimaliaHymenopteraIchneumonidae

(Cameron, 1907)

Megatrema albopilosa Cameron 1907: 468. Type: male; Type depository: NHM.
Seticornuta albicalcar Morley 1913: 310. Type: female; Type depository: ZSI.

#### Redescription based on holotype.

**Male.** Fore wing 10 mm, body 13.0 mm.

#### Color.

Black. Antennal scape and basal 1–14 flagellomeres reddish brown; wings dark brown; fore leg reddish brown, mid and hind legs blackish brown; tegula dark brown.

#### Morphology.

***Head*.** Face swollen, 1.2 times as long as wide in frontal view. Occipital carina strong from above and laterally. Frons with moderately coarse and dense punctures. Diameter of lateral ocellus equal to distance between ocellus and eye. Flagellum thickened in basal half, tapered to apex, with 42 + (antenna broken) flagellomeres. First flagellomere as long as wide, next flagellomere transverse and last several flagellomeres square. Clypeus not separated from face. Face moderately punctate, distance between punctures equal to their diameter. Malar space 0.6 times as long as basal width of mandible. Gena acuminate in frontal view (Fig. 3D, dotted line and arrow).

***Mesosoma*.** Flattened, 2.0 times as long as high in lateral view. Lower part of pronotum smooth, impunctate, epomia weak. Pronotum rounded in dorsal view (Fig. 3E, dotted line and arrow). Mesoscutum elongate with sparse punctures, rounded in lateral view (Fig. 3F, dotted line and arrow), notaulus weak. Scutellum flat with lateral carinae. Epicnemial carina strong, reaching subtegular ridge. Areolet of fore wing present, with stalk shorter than vein 2rs-m; fore wing vein cu-a curved (Fig. 3H). Hind wing with 11 distal hamuli. Nervellus inclivous, intercepted distinctly below middle. Legs very stout. Hind femur coarsely punctate, 3.0 times as long as wide. Spurs of mid leg with of equal length. Tarsomeres 2–4 of fore leg shorter than wide. Propodeum with very strong median longitudinal and apical transverse carinae. Combined basal area and area superomedia convergent apically. Basal area not separated from carina. Costula absent. Propodeal spiracle 3.7 times as long as wide, joining pleural carina.

***Metasoma*.** Median dorsal carinae of first tergite very strong, extending to 2/3 its length. Second tergite 0.7 times as long as wide. Metasoma covered with rather long hairs.

#### Material examined.

Holotype: male of *Megatrema albopilosa* (NHM) (Fig. 2).

#### Distribution.

Eastern Palaearctic and Oriental regions: China (Henan), India, Myanmar, Sri Lanka.

#### Host.

Unknown.

## Supplementary Material

XML Treatment for Seticornuta

XML Treatment for Seticornuta koreana

XML Treatment for Seticornuta albopilosa
